# Intracellular glutathione determines bortezomib cytotoxicity in multiple myeloma cells

**DOI:** 10.1038/bcj.2016.56

**Published:** 2016-07-15

**Authors:** K K Starheim, T Holien, K Misund, I Johansson, K A Baranowska, A-M Sponaas, H Hella, G Buene, A Waage, A Sundan, G Bjørkøy

**Affiliations:** 1 Department of Cancer Research and Molecular Medicine, K.G. Jebsen Center for Myeloma Research, Norwegian University of Science and Technology, Trondheim, Norway; 2 Department of Cancer Research and Molecular Medicine, Center of Molecular Inflammation Research, Norwegian University of Science and Technology, Trondheim, Norway; 3 Department of Laboratory Medicine, Children's and Women's Health, Faculty of Medicine, Norwegian University of Science and Technology, Trondheim, Norway; 4 Department of Hematology, St. Olavs University Hospital, Trondheim, Norway; 5 Department of Medical Laboratory Technology, Faculty of Technology, Norwegian University of Science and Technology, Trondheim, Norway

## Abstract

Multiple myeloma (myeloma in short) is an incurable cancer of antibody-producing plasma cells that comprise 13% of all hematological malignancies. The proteasome inhibitor bortezomib has improved treatment significantly, but inherent and acquired resistance to the drug remains a problem. We here show that bortezomib-induced cytotoxicity was completely dampened when cells were supplemented with cysteine or its derivative, glutathione (GSH) in ANBL-6 and INA-6 myeloma cell lines. GSH is a major component of the antioxidative defense in eukaryotic cells. Increasing intracellular GSH levels fully abolished bortezomib-induced cytotoxicity and transcriptional changes. Elevated intracellular GSH levels blocked bortezomib-induced nuclear factor erythroid 2-related factor 2 (NFE2L2, NRF2)-associated stress responses, including upregulation of the xCT subunit of the Xc- cystine-glutamate antiporter. INA-6 cells conditioned to increasing bortezomib doses displayed reduced bortezomib sensitivity and elevated xCT levels. Inhibiting Xc- activity potentiated bortezomib-induced cytotoxicity in myeloma cell lines and primary cells, and re-established sensitivity to bortezomib in bortezomib-conditioned cells. We propose that intracellular GSH level is the main determinant of bortezomib-induced cytotoxicity in a subset of myeloma cells, and that combined targeting of the proteasome and the Xc- cystine-glutamate antiporter can circumvent bortezomib resistance.

## Introduction

Multiple myeloma is a cancer resulting from the malignant transformation and clonal expansion of antibody-producing plasma cells.^1^ It is the second most common form of hematological cancers in Western countries, and it makes up 1% of all cancer deaths in the United States. As for today, myeloma is incurable with a median survival of 5–7 years from time of diagnosis. Response to treatment is variable, reflecting the diverse genetic events causing myeloma.^2^

Drugs targeting the ubiquitin-proteasome system have drastically improved myeloma treatment. The proteasome inhibitor bortezomib inhibits proteasome-mediated protein degradation by binding to the site with chymotrypsin-like activity on the 26S proteasome.^3^ Blocking proteasomal degradation leads to the accumulation of intracellular proteins and subsequent cellular stress,^4^ which in turn inhibits proliferation and induces apoptosis of myeloma cells.^5, 6^ Through their role as antibody producers myeloma cells have a high protein synthesis rate, and this might render them more vulnerable to disturbances in the protein degradation machinery.^7^ However, inherent and acquired resistance toward proteasome inhibitors remains a problem. The mechanisms behind clinical resistance to proteasomal inhibitors remain elusive.^3^

Depletion of glutathione (γ-glutamylcysteinylglycine, GSH) can potentiate the effect of bortezomib in myeloma cells.^8^ GSH is an important buffering agent that maintains redox homeostasis in eukaryotic cells,^9^ and increased GSH levels are associated with cancer development.^10^ GSH is made *de novo* and the rate-limiting substrate in this process is cysteine.^9^ Hematopoietic cells do not synthesize cysteine, and are therefore dependent on uptake of circulating cystine.^11^ The cystine-glutamate antiporter Xc- provides cellular cysteine supply, and is thus an important regulator of intracellular GSH synthesis. Xc- imports cystine into the cytoplasm, where it is quickly reduced to cysteine. Xc- consists of the light-chain subunit xCT (SLC7A11) and a heavy-chain subunit (4F2hc/SLC3A2).^11^ xCT expression is increased in several cancers, and is associated with drug resistance and poor survival.^12, 13, 14, 15^

One of the major regulators of cellular stress response upstream of GSH is nuclear factor erythroid 2-related factor 2 (NFE2L2, NRF2).^11, 16^ Deregulation of the NFE2L2-mediated stress-protection program has emerged as central in the development of several cancer types.^17, 18^ As a response to various stressors, NFE2L2 translocates to the nucleus for activation of stress-protective genes, including *NAD(P)H dehydrogenase, quinone 1* (*NQO1*)*, heme oxygenase 1* (*HMOX1*), *sequestosome 1* (*SQSTM1, P62*) and *SLC7A11*.^16, 19^ *SQSTM1* encodes the autophagy cargo receptor protein sequestosome 1 (SQSTM1/p62), which selectively collects ubiquitinated protein to aggregates.^20, 21^ Accumulation of ubiquitinated misfolded proteins leads to SQSTM1-mediated activation of NFE2L2.^22, 23, 24^

Here, we show that increased supply of GSH and cysteine elevates intracellular GSH levels and neutralizes bortezomib-induced cytotoxicity in a subset of myeloma cell lines. Reciprocally, decreasing intracellular GSH levels through blocking the Xc-cystine-glutamate antiporter subunit xCT potentiated bortezomib-induced cytotoxicty in myeloma cell lines and primary cells. Xc- represents a potential target for therapy in combination with bortezomib in myeloma patients.

## Materials and methods

### Cell culture

The myeloma cell lines used in this study were ANBL-6 (a kind gift from Dr Diane Jelinek, Mayo Clinic, Rochester, MN, USA), INA-6 (a kind gift from Dr Martin Gramatzki, Erlangen, Germany^25^), U266 (ATCC, Rockville, MD, USA), IH-1,^26^ OH-2,^27^ JJN3 (a kind gift from Dr Jennifer Ball, Department of Immunology, University of Birmingham, UK) and KJON (established in our laboratory). ANBL-6 and INA-6 cells were grown in 10% heat-inactivated fetal calf serum in RPMI-1640 (hereafter described as RPMI, Sigma-Aldrich, St. Louis, MO, USA, R8758) supplemented with recombinant human interleukin (IL)-6 (1 ng/ml) (Gibco, Life Technologies/Thermo Fisher, Waltham, MA, USA). U266 cells were maintained in 15% fetal calf serum in RPMI. IH-1 and OH-2 cells were maintained in 10% heat-inactivated human serum (HS, Blood Bank, St Olav's University Hospital, Trondheim, Norway) in RPMI and IL-6 (2 ng/ml). JJN3 cells were maintained in 10% heat-inactivated fetal calf serum in RPMI-1640. KJON cells were maintained in RPMI with 5% HS and IL-6 (2 ng/ml). All cells were cultured at 37 °C in a humidified atmosphere containing 5% CO_2_. For bortezomib conditioning, INA-6 cells were cultivated in media with an initial dose of 2 nm bortezomib. Cells were split twice every week, and the bortezomib dose was gradually increased with about 1 nm/month to a final dose of 9 nm after 6 months. Cells were then maintained in 9 nm bortezomib for 3 months, and then maintained in bortezomib-free medium for 4 weeks before being subjected to experiments. Unconditioned cells were maintained in parallel, by the same schedule. To obtain primary myeloma cells, CD138+ cells were isolated from bone marrow specimens obtained through the Norwegian Myeloma Biobank using RoboSep automated cell separator and Human CD138 Positive Selection Kit (StemCell Technologies, Grenoble, France). Informed consent was obtained from participating patients, and the regional ethics committee approved the study (REK Midt 2011/2029).

### Reagents

The following antibodies were used: β-actin/ACTB (Abcam, Cambridge, UK, #6276), GAPDH (Abcam, #8245), MAP1LC3B/LC3B (Cell Signaling, Beverly, MA, USA, #3868), HMOX1 (Enzo, Farmingdale, NY, USA, #ADI-OSA-110), IgG Fcγ fragment specific (Jackson ImmunoResearch Laboratories, West Grove, PA, USA, #109-006-098), FK2 antibody recognizing mono- and polyubiquitinated proteins (Enzo, #BML-PW-8810), SQSTM1 (Progen, Heidelberg, Germany, #GP62-C), SRXN1 (Proteintech, Chicago, IL, USA, #14273-1-AP), xCT (Cell Signaling, Beverly, MA, USA, #12691). As bortezomib-induced cytotoxicity varied between batches and storage time, bortezomib (Selleck Chemicals, Houston, TX, USA) toxicity was titrated for each batch used, and regularly controlled. Supplements of 1 mm cysteine, GSH and alanine, or 5 mm glutamate (Sigma-Aldrich) were given for 24 h. Where indicated, minimal essential medium (Gibco/Thermo Fisher Scientific, Waltham, MA, USA, #11130-036) was added to a final 1 × concentration to restore levels of essential amino acids, including l-cystine. Sulfasalazine (Fluka/Sigma-Aldrich) was used as indicated. Dimethyl sulfoxide (DMSO) was from Sigma-Aldrich. Hydrogen peroxide was from Merck Millipore (#1.08600, Billerica, MA, USA).

### Cell lysis and immunoblotting

Cells were sedimented and re-suspended in an 8-m Urea and 0.5% Triton X-100 lysis solution and sonicated 4 × for 1s. Lithium dodecyl sulfate sample buffer (Life Technologies/Thermo Fisher, Waltham, MA, USA) with dithiothreitol reducing agent (final concentration of 25 mm) was added to cell lysates before heating to denature proteins. Reduced samples were electrophoresed using 4–12% NuPAGE Bis-Tris gels and MOPS running buffer (Life Technologies). Proteins were transferred from the gel onto a 0.2 μm nitrocellulose membrane using the iBlot gel transfer system (Life Technologies). The membranes were blocked with 50% Odyssey Blocking Buffer (LI-COR, Lincoln, NE, USA) in TBS containing 0.1% Tween-20, and incubated overnight with primary antibodies. The blots were developed using IRDye (LI-COR) or HRP-conjugated secondary antibodies (DakoCytomation, Glostrup, Denmark) and visualized with LI-COR Odyssey Imager. LiCor Image Studio was used for immunoblot quantification.

### Apoptosis measurement

The extent of apoptosis was measured by flow cytometry using the APOTEST-FITC Kit according to the manufacturer's instructions (Nexins Research, Kattendijke, The Netherlands). Cells negative for Annexin-V and propidium iodide were counted as viable. For the ScanR Cell Viability Assay, myeloma cell lines or primary myeloma cells were plated in 96-well plates, treated as indicated, and cell death was measured as described previously.^28^

### Gene expression analysis

Total RNA was isolated using the High Pure RNA Isolation Kit (Roche, Basel, Switzerland), and complementary DNA was synthesized using the iScript Select cDNA synthesis kit (170-8896, Bio-Rad, Hercules, CA, USA). Microarray analysis was performed using the Illumina Gene Expression Array platform. Data are available in the ArrayExpress database (www.ebi.ac.uk/arrayexpress) under accession number E-MTAB-2666. Real-time PCR was performed in parallel in 25 μl reactions containing 12.5 μl 2 × QuantiTect SYBR Green PCR master mix (Qiagen, Venlo, The Netherlands) and 2.5 μl 10 × QuantiTect Primer Assay (Qiagen). The following primers were used: *ACTB* (Sigma-Aldrich) fwd. cat. no. 8008077200-000030, rev. cat. no. 8008077200-000040; *GAPDH* (Thermo Fisher) primer set cat. no. Hs-99999905_m1; *HMOX1* (Sigma-Aldrich) fwd. cat. no. 8011377832-000090, rev. cat. no. 8011377832-000100; *NQO1* (Sigma-Aldrich) fwd. cat. no. 8011377832-000010, rev: 8011377832-000020; *SLC7A11* (Thermo Fisher) primer set cat. no. Hs01099013_m1. Relative RNA transcription levels were transformed into a linear form by 2-deltadeltaCT using *ACTB* or *GAPDH* as housekeeping genes.

### Proteasome activity

Proteasome activity was measured using the Proteasome-Glo Chymotrypsin-Like Cell-Based Assay (Promega, Madison, WI, USA) according to the manufacturer's instructions. 20S proteasomes isolated from human erythrocytes (Boston Biochemicals, Cambridge, MA, USA) were used for all proteasome activity assays.

### GSH levels

GSH levels were measured using the GSH-GLO Glutathione Assay (Promega) according to the manufacturer's instructions.

### CRISPR/Cas9 gene editing

INA-6 cells were transduced with lentiviral CRISPR/Cas9 particles targeting *SLC7A11* (exon 1, HS0000361320) or non-targeting control particles (Sigma-Aldrich) using a multiplicity of infection of 10. The cells were then grown in the presence of puromycin to select for positive clones. Limited dilution of puromycin-selected cells was performed to acquire single cell clones. None of the final clones had complete knockout, as determined by immunoblotting. We picked two clones with partial knockout as well as two control clones transduced with non-targeting particles for use in experiments.

### Statistical analysis

Statistical analysis was performed using the GraphPad Prism 6 software (GraphPad Software Inc., La Jolla, CA, USA). Student's *t*-test was used for comparison in setups with two groups. A two-way between groups analysis of variance (ANOVA) with a Turkey's multiple comparisons test was used for comparison between groups in multi-group setups. For dose–response curves, significant differences in response were calculated as follows: data were normalized within groups and IC50 was calculated by linear regression (non-linear regression, log(inhibitor) vs response, variable slope). Extra sum-of-squares F-test was used to test whether IC50 values differed between groups. Level of statistical significance was set at 0.05 (5%) for all experiments.

## Results

### Cysteine and GSH supplement abolished bortezomib-induced cytotoxicity in several myeloma cell lines

**Figure 1:**
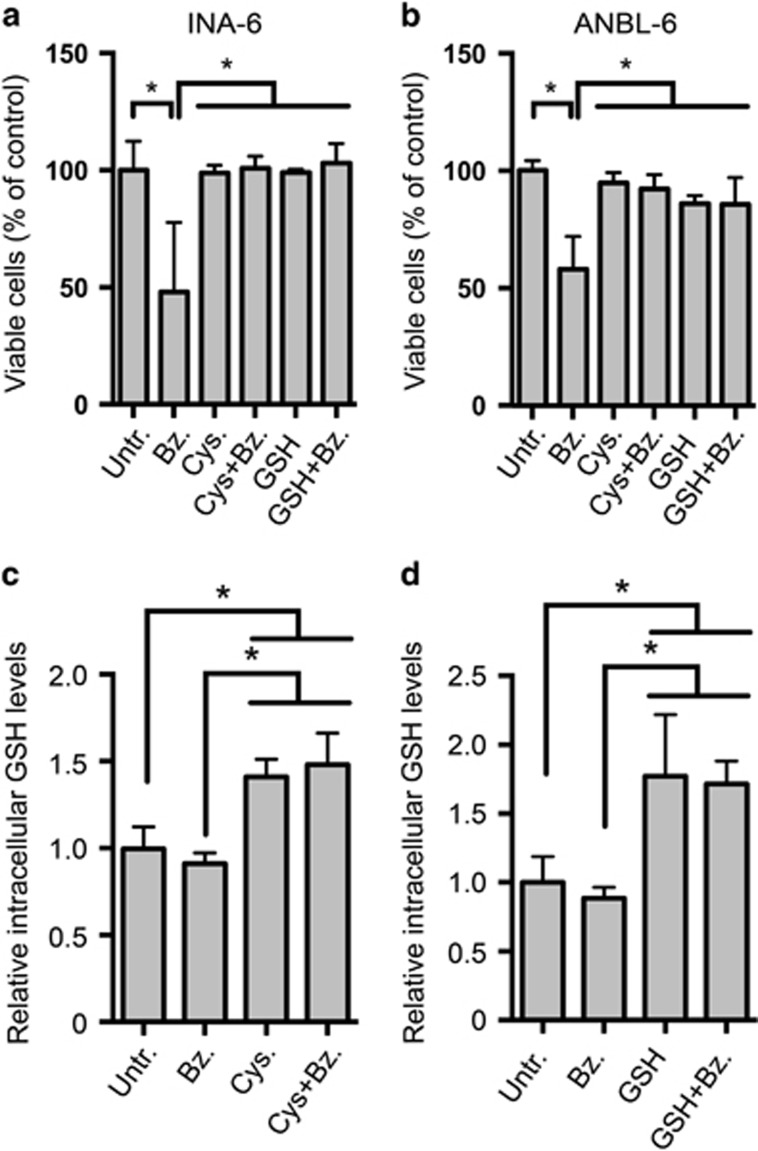
Cysteine and GSH supplements dampened bortezomib-induced cytotoxicity and increased intracellular GSH levels in myeloma cell lines. INA-6 (**a**) or ANBL-6 cells (**b**) were treated with 4 nm bortezomib (Bz) for 24 h in the presence of 1 mm cysteine (Cys) or GSH as indicated. Cell viability was measured using Annexin V-propidium iodide (PI) staining and flow cytometry as described in Materials and methods. INA-6 cells were treated with 5.5 nM bortezomib in combination with 1 mm cysteine (**c**) or GSH (**d**) as in (**a**). Intracellular GSH concentrations were measured as described in Materials and methods. Data are mean and s.d. for at least three independent experiments. Asterisks indicate statistically significant differences (two-way ANOVA, Turkey's multiple comparisons test, *P*<0.05).

It was recently shown that amino-acid depletion was the cause of bortezomib-induced cell death in several eukaryotic systems.^29^ To investigate whether amino-acid supplements could counter bortezomib-induced cytotoxicity, we supplied INA-6 and ANBL-6 myeloma cell lines with alanine, cysteine or a mixture of amino acids in minimal essential medium. Supplementing the cell lines with alanine or minimal essential medium had no effect on viability in bortezomib-treated INA-6 or ANBL-6 cells (Supplementary Figure S1A). In contrast, cysteine supplements dampened the cytotoxic effects of bortezomib in INA-6 and ANBL-6 cells (Figures 1a and b, Supplementary Figure S1A). Restricting amino-acid availability by growing cells in 50% diluted medium (1:1 RPMI:Hank's Balanced Salt Solution, HBSS) potentiated bortezomib-induced cytotoxicity, and also this was dampened by cysteine (Supplementary Figure S1B, C). Cysteine dampened bortezomib cytotoxicity also in JJN3 cells but not in U266, IH-1 or OH-2 cells (Supplementary Figure S1C).

Cysteine is an essential amino acid for hematopoietic cells, and it is the rate-limiting substrate in GSH synthesis.^11^ GSH supplements also dampened bortezomib-induced cytotoxicity in INA-6 and ANBL-6 cells (Figures 1a and b). Cysteine and GSH supplements significantly increased intracellular GSH levels in untreated and bortezomib-treated cells (Figures 1c and d). Cysteine or GSH supplements did not interfere with bortezomib-mediated inhibition of 20S proteasomes, as measured by the Proteasome-Glo Chymotrypsin-Like Cell-Based Assay (data not shown). Thus, cysteine and GSH likely does not interfere with bortezomib-mediated proteasome inhibition. These data suggest that in myeloma cells, bortezomib-induced cell death is not mediated by a general lack of amino acids but rather due to limited levels of intracellular GSH.

### Bortezomib activated the NFE2L2-dependent stress response and GSH-production machinery

**Figure 2:**
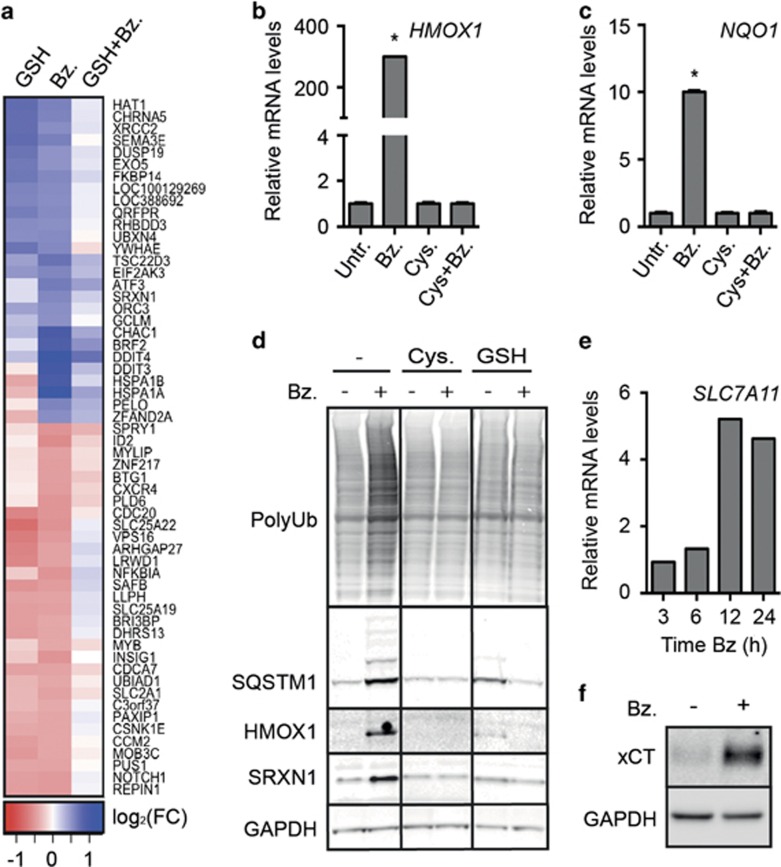
Cysteine and GSH attenuated bortezomib-induced transcriptional changes, protein ubiquitination and upregulation of redox-defense proteins. (**a**) INA-6 cells were grown in RPMI, diluted to 50% in HBSS and treated with 4 nm bortezomib (Bz), 1 mm GSH, or a combination of bortezomib and GSH for 4 h. RNA was collected and analyzed by Illumina Gene Expression assay. The heatmap displays genes differentially expressed after bortezomib treatment, and dampened by the combination (bz/DMSO<0.05 and bz/bz+GSH<0.05). The heatmap was generated from log FC ratios, with a ratio cutoff of >0.5 (upregulated genes) or >0.4 (downregulated genes) (see Supplementary Table S1). The experiment was performed in triplicates. (**b**, **c**) INA-6 cells were treated with 4 nm bortezomib with or without 1 mm cysteine (Cys) supplement for 4 h. RNA was collected and analyzed for mRNA levels of *HMOX1* (**b**) or *NQO1* (**c**) by qPCR. Data are mean and s.d. for triplicates in one representative experiment of at least three independent experiments. Asterisks indicate statistically significant changes compared with the control (two-way ANOVA, Turkey's multiple comparisons test, *P*<0.05). (**d**) INA-6 cells were treated for 24 h with 4 nm bortezomib in the presence of 1 mm cysteine or GSH. Cells were lysed and protein levels were analyzed by immunoblotting, as indicated. GAPDH was used as a loading control. (**e**) INA-6 cells were treated with 5 nm bortezomib for the indicated time points. RNA was collected and analyzed for mRNA levels of *SLC7A11* by qPCR. (**f**) INA-6 cells were treated with 4 nm bortezomib for 24 h. Cells were lysed and xCT protein levels were analyzed by immunoblotting. GAPDH was used as a loading control. Data are representative for at least three independent experiments (**d**–**f**).

Gene expression profiling was performed to determine which subcellular processes that may be involved in GSH-mediated protection toward bortezomib-induced cytotoxicity. INA-6 cells cultured with or without GSH were treated with bortezomib before analysis of gene expression by the Illumina Gene Expression Array platform. Bortezomib treatment of cells resulted in marked changes in gene expression. Interestingly, although GSH treatment alone also invoked some gene expression changes, GSH supplements dampened most of the bortezomib-induced gene expression changes (Figure 2a). Specifically, 152 genes displayed a bortezomib-induced upregulation that was significantly neutralized in the presence of GSH (*P*<0.05) (Supplementary Table S1A), while 146 genes displayed a bortezomib-induced downregulation, which was significantly dampened by GSH (Supplementary Table S1B). The upregulated genes were involved in endoplasmic reticulum stress, unfolded protein response and oxidative stress, while genes involved in anabolic processes were downregulated in response to bortezomib in a GSH-dependent manner (Figure 2a, Supplementary Table S2).

The gene expression data demonstrated that several NFE2L2 target genes were upregulated in bortezomib-treated cells, including *SRNX1* and *GCLM,* encoding sulfiredoxin-1 (SRXN1) and glutamate-cysteine ligase modifier subunit (GCLM), respectively (Supplementary Table S1).^30, 31^ Consistently, qPCR analyses revealed that the NFE2L2 target genes *HMOX1* and *NQO1* were significantly upregulated in bortezomib-treated cells in a cysteine-dependent manner (Figures 2b and c).

On the protein level, bortezomib treatment induced HMOX1, SRXN1 and SQSTM1 as well as ubiquitin smears. Cysteine or GSH abolished these effects (Figure 2d). In addition to the increased protein level of SQSTM1, bortezomib treatment also caused formation of larger, modified forms of SQSTM1. GSH supplements alone also induced somewhat higher levels of SQSTM1, HMOX1 and SRXN1, indicating that regulating GSH levels in redox homeostasis is a fine-tuned process. Consistent with NFE2L2 activation, bortezomib also induced increased mRNA level of *SLC7A11* as well as the protein level of the corresponding xCT subunit of the Xc- antiporter (Figures 2e and f).

In summary, bortezomib induced major transcriptional changes, including the NFE2L2-dependent oxidative stress responsive cystein import system, whereas cysteine and GSH supplements dampened these changes.

### Bortezomib-conditioned cells displayed increased xCT levels

**Figure 3:**
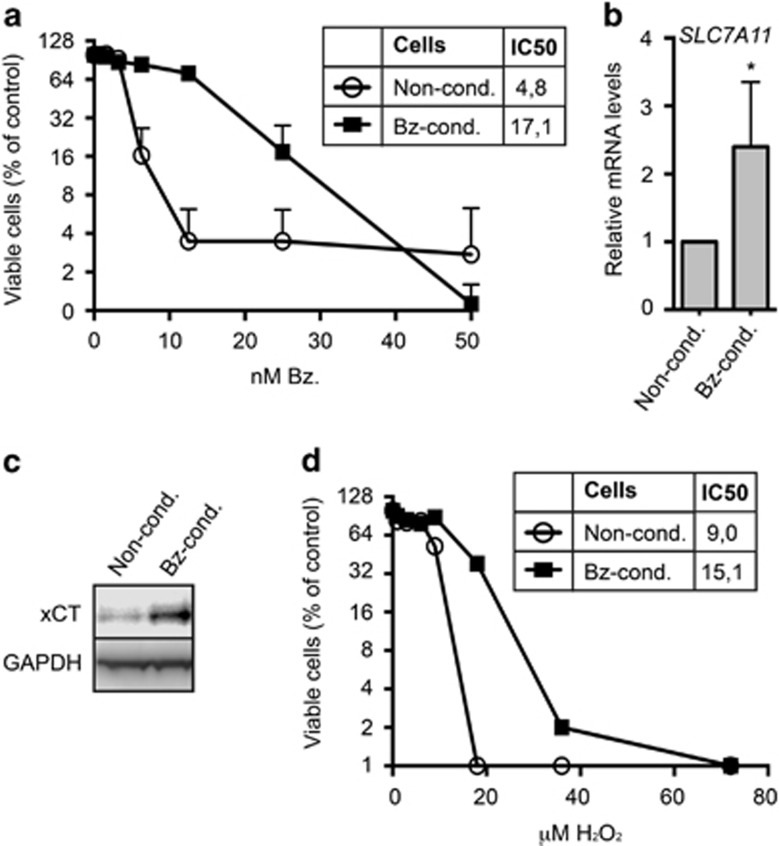
Bortezomib conditioning reduced bortezomib sensitivity and increased xCT and GSH levels in INA-6 cells. (**a**) Bortezomib-conditioned (Bz-cond.) or non-conditioned (Non-cond.) INA-6 cells were treated with the indicated doses of bortezomib (Bz.) for 24 h. Cell death was measured using the ScanR microscope, as described in Materials and methods. The results represent the mean and s.d. of three independent experiments. Bortezomib conditioning significantly increased the IC50 (nm) of bortezomib. (**b**) RNA was collected from bortezomib-conditioned and non-conditioned INA-6 cells and analyzed for mRNA levels of *SLC7A11* by qPCR. Data are mean and s.d. from three independent experiments. The asterisk indicates a statistically significant difference as compared with the control (*P*<0.05, Student's *t*-test). (**c**) Bortezomib-conditioned and non-conditioned INA-6 cells were lysed and baseline xCT levels were analyzed by immunoblotting. GAPDH was used as a loading control. Data are representative for at least three independent experiments. (**d**) Bortezomib-conditioned and non-conditioned INA-6 cells were treated with the indicated doses of hydrogen peroxide (H_2_O_2_) for 16 h. Cell death was measured using the ScanR microscope. Bortezomib conditioning significantly increased the IC50 (μm) of hydrogen peroxide. Data display mean and s.d. of duplicates from one representative of three independent experiments. (**a**, **d**) IC50 was calculated using non-linear regression. Extra sum-of-squares F-test was used to test for significant shifts in IC50 (*P*<0.05).

We hypothesized that myeloma cells could decrease their sensitivity to bortezomib through upregulation of the cystine import system. To test this, we cultured INA-6 cells in increasing concentrations of bortezomib. The cells displayed an increased tolerance for bortezomib as the dose was gradually increased. Bortezomib exposure was then ended, and cells were maintained in bortezomib-free medium. After about 8 weeks post bortezomib exposure, bortezomib-conditioned cells still displayed significantly lower sensitivity to bortezomib as compared with non-conditioned INA-6 cells (Figure 3a). Furthermore, the conditioned cells displayed higher levels of *SL7A11* mRNA and xCT protein as compared with the non-conditioned cells (Figures 3b and c). Bortezomib-conditioned cells also displayed higher tolerance toward hydrogen peroxide (Figure 3d), suggesting that they are more capable of handling oxidative stress in general, as compared with non-conditioned cells.

### Inhibition of xCT potentiated bortezomib-induced cytotoxicity

**Figure 4:**
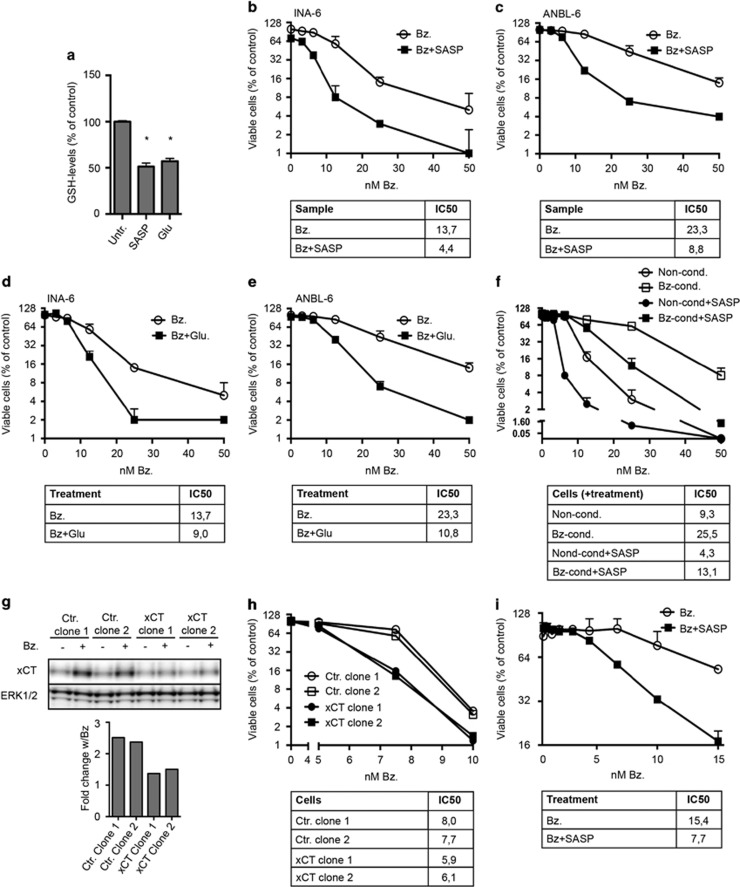
Inhibiting Xc- activity reduced intracellular GSH levels and potentiated bortezomib-induced cytotoxicity in myeloma cell lines and primary cells. (**a**) ANBL-6 cells were treated with 0.25 mm sulfasalazine (SASP) or 5 mm glutamate (Glu) for 16 h. Intracellular GSH was measured as described in Materials and methods. Data are mean and s.d. from triplicates of one representative of three independent experiments. Asterisk indicates statistically significant differences as compared with the control (two-way ANOVA, Turkey's multiple comparisons test, *P*<0.05). INA-6 (**b**) or ANBL-6 cells (**c**) were treated with the indicated doses of bortezomib (Bz) alone or in combination with 0.25 mm sulfasalazine for 24 h. Sulfasalazine treatment induced a significant decrease in the IC50 of bortezomib. INA-6 (**d**) or ANBL-6 cells (**e**) were treated with the indicated doses of bortezomib and 5 mm glutamate for 24 h. Glutamate treatment induced a significant decrease in IC50 of bortezomib. (**f**) Bortezomib-conditioned (Bz-cond.) and non-conditioned (Non-cond.) INA-6 cells were treated and analyzed as in (**b**). Sulfasalazine treatment induced a significant decrease in IC50 of bortezomib in both conditioned and non-conditioned cells. (**g**) *SLC7A11* was targeted in INA-6 cells using CRISPR/Cas9. Clones were lysed and xCT levels were determined by immunoblotting. Bars give the quantified bortezomib-induced change of xCT levels. ERK1/2 was used as a loading control as GAPDH levels were reduced in xCT clones. Data are representative of at least three independent experiments. (**h**) Clones from (**g**) were treated with the indicated doses of bortezomib and 0.25 mm sulfasalazine for 24 h. Bortezomib displayed a significantly lower IC50 in xCT knockdown clones than in non-targeting control clones. (**i**) CD138^+^ plasma cells from a myeloma patient were treated for 3 days with bortezomib alone or in combination with 0.25 mm sulfasalazine. Sulfasalazine treatment induced a significant decrease in IC50 of bortezomib. The ScanR microscope was used to measure cell death. All IC50 values are in nm, and were calculated using non-linear regression. Extra sum-of-squares F-test was used to test for significant shifts in IC50 (*P*<0.05). (**b–f**) and (**h**) Mean and standard deviation from duplicates in one representative experiment of at least three independent experiments is shown. (**i**) Mean and standard deviation from duplicates in one experiment is shown.

We hypothesized that the activity of the Xc- cystine-glutamate antiporter could influence the cellular sensitivity to bortezomib. To investigate this, we blocked Xc- activity by using the Xc- specific inhibitor sulfasalazine and through blocking the Xc- cystine-glutamate antiporter activity by addition of extracellular glutamate.^11^ Both sulfasalazine and glutamate treatment reduced intracellular GSH levels in ANBL-6 cells (Figure 4a) and potentiated bortezomib-induced cytotoxicity in INA-6 and ANBL-6 cells (Figures 4b–e). Sulfasalazine treatment also increased bortezomib sensitivity in bortezomib-conditioned cells (Figure 4f). To further investigate the role of Xc- in bortezomib sensitivity, we targeted the *SLC7A11* gene by CRISPR-Cas9 to knockout xCT expression. In line with an essential role of xCT, the obtained clones displayed only partial reduction in the xCT protein level. However, the xCT clones were significantly more sensitive to bortezomib than the control clones and they did not mobilize xCT protein in response to bortezomib (Figures 4g and h).

Collectively, these cell line-based experiments suggest an inverse correlation between the xCT protein level and sensitivity for bortezomib. We then asked whether this could be observed also in patients displaying different sensitivity for the drug, However, we could not detect any clear correlation between bortezomib sensitivity and *SLC7A11* mRNA levels in isolated primary myeloma cells isolated from BM of sensitive and clinically resistant patients (*n*=10 for each group, results not shown). We then inhibited cysteine uptake in primary cells using sulfasalazine and asked whether this changed the response to the bortezomib. Primary myeloma isolates from four different patients were treated with the indicated doses of bortezomib in combination with sulfasalazine. Sulfasalazine potentiated bortezomib cytotoxicity in three of four tested isolates (Figure 4i, Supplementary Figure S2).

In summary, inhibiting xCT activity reduces intracellular GSH levels and potentiates bortezomib-induced cytotoxicity in myeloma cells.

## Discussion

In this study, we present findings showing that intracellular GSH level is a major determinant of bortezomib-induced cytotoxicity. GSH and cysteine supplements can to a large extent neutralize bortezomib-induced cytotoxicity in myeloma cells, and bortezomib-conditioned cells display increased levels of xCT. Inhibiting cystine importer Xc- activity decreases intracellular GSH levels and increases bortezomib cytotoxicity in both myeloma cell lines and primary cells.

The mechanism of bortezomib-induced cytotoxicity has remained elusive. It was recently suggested that bortezomib induced cytotoxicity through amino-acid depletion.^29^ We found that cysteine and GSH rather than amino acids in general attenuated bortezomib-induced cell death in myeloma cell lines. This is consistent with earlier studies showing that the effects of bortezomib correlate with intracellular GSH levels in myeloma cells.^8^ We here extend those findings by showing that GSH levels can broadly neutralize bortezomib-induced cytotoxicity, and that the cystine-glutamate antiporter Xc- is central in regulating intracellular GSH levels and therefore bortezomib sensitivity of myeloma cells. As hematopoetic cells do not produce cysteine,^11^ this can explain the important role of Xc- for bortezomib-mediated cytotoxicity in myeloma cells. However, myeloma development is a complex and heterogenic process. In this work, the genetic heterogeneity of myeloma cells is reflected in the bortezomib sensitivity, both in patients and in myeloma cell lines. The reasons for these variations are unclear and likely complex, but several stress-handling mechanisms such as autophagy and other ROS scavenging mechanisms may play a part.

Cysteine is imported in the form of cystine through the cystine-glutamate antiporter Xc-.^11^ Both acute cytotoxic bortezomib treatment and low-grade sub-lethal bortezomib treatment induced an increase in xCT levels in myeloma cell lines. Blocking Xc- activity either through inhibition or xCT depletion increased bortezomib sensitivity and restored sensitivity in bortezomib-conditioned cells. This shows that the broad effect of cysteine on bortezomib cytotoxicity to a large extent can be accounted for by Xc-.

INA-6 cultures grown in sub-lethal and step-wise increased doses of bortezomib displayed decreased bortezomib sensitivity even 8 weeks after bortezomib conditioning was terminated. This suggests a stable genetic or robust epigenetic change in the INA-6 culture, possibly through gradual selection. Bortezomib-conditioned cells displayed higher levels of *SLC7A11* and xCT than non-conditioned cells. We hypothesize that clonal evolution during bortezomib treatment can lead to Xc- and GSH-mediated bortezomib resistance *in vivo.* We investigated whether xCT mRNA levels could be used as a biomarker for bortezomib sensitivity in myeloma patients but no clear correlation was found for the 20 patients analyzed. Developing methods that could provide a reliable readout for Xc- activity in primary myeloma cells could contribute to a more personalized use of proteasomal inhibitors in myeloma patients.

Myeloma cells produce high levels of immunoglobulins. They might rely on an efficient GSH system to reduce protein misfolding due to ROS and to handle toxic protein derivatives, in addition to a well-functioning proteasomal system for efficient turnover of misfolded proteins. We suggest that myeloma cells can counter bortezomib-induced cellular stress through increasing their ability to regulate intracellular GSH levels. This can create a more robust intracellular homeostasis and decrease the amount of misfolded proteins that are ubiquitinated and need to be cleared by the ubiquitin-proteasome system.

Bortezomib treatment did not reduce GSH levels, but cysteine and GSH supplements increased intracellular GSH levels. The striking attenuation by GSH on bortezomib-induced cytotoxicity was also reflected in gene expression levels of INA-6 cells. The global reversion of bortezomib-induced transcriptional changes indicated that GSH directly neutralized the cytotoxic processes resulting from bortezomib treatment rather than affecting signaling downstream of these processes. The level of ubiquitinated proteins accumulating after bortezomib treatment was also dampened by cysteine and GSH supplements. Neither cysteine nor GSH interfered with bortezomib-induced proteasome inhibition. Other work from our laboratory shows that there is no compensatory upregulation of autophagy in bortezomib-treated cells (Baranowska *et al.*, submitted manuscript). Thus, cysteine and GSH decrease the general level of protein ubiquitination.

A puzzling observation was that although intracellular GSH levels are dependent on cysteine availability, GSH supplements also dampened bortezomib cytotoxicity. We speculate that GSH is converted to cystine extracellularly, as is the case with cysteine, or that GSH can be directly imported into the cells through mechanisms independent of Xc-.

In summary, our work warrants clinical studies combining bortezomib with drugs that reduce the intracellular GSH levels. Targeting cystine uptake has been suggested as therapy of several different cancers.^14^ Sulfasalazine may be one possibility as this drug is clinically approved and has systemic effects *in vivo*.^32^ However, as sulfasalazine is metabolized to 5-acylsalisylic acid and sulfapyridine in the gut,^32^ pharmacodynamics will be more complex in a clinical setting. Further work is needed to determine whether sulfasalazine is a feasible candidate for combination therapy with bortezomib in multiple myeloma patients.
