# Supplementary material for: Intracellular glutathione determines bortezomib cytotoxicity in multiple myeloma cells
Source: Blood Cancer J. 2016 Jul 15;6(7):e446–. doi: 10.1038/bcj.2016.56 (PMC5141348; doi:10.1038/bcj.2016.56)
Supplement: Supplementary Information [file bcj201656x1.doc]

**Supplementary data for**

**INTRACELLULAR GLUTATHIONE DETERMINES BORTEZOMIB CYTOTOXICITY IN MULTIPLE MYELOMA CELLS**.

Kristian K. Starheim^1,2^, Toril Holien^1^, Kristine Misund^1^, Ida Johansson^2,4^, Katarzyna A. Baranowska^1^, Anne-Marit Sponaas^1^, Hanne Hella^1^, Glenn Buene^1^, Anders Waage^1, 3^, Anders Sundan^1, 2^, Geir Bjørkøy ^1,2^.

^1^KG. Jebsen Center for Myeloma Research, Department of Ca

ncer Research and Molecular Medicine, Norwegian University of Science and Technology, Trondheim N-7491, Norway; ^2^Center of Molecular Inflammation Research, Department of Cancer Research and Molecular Medicine, Norwegian University of Science and Technology, Trondheim N-7491, Norway; ^3^Department of Hematology, St. Olavs University Hospital, Trondheim, Norway; ^4^Department of Laboratory Medicine, Children’s and Women’s Health, Faculty of Medicine, Norwegian University of Science and Technology, Trondheim, Norway

**Correspondence to**: Geir Bjørkøy. Norwegian University of Science and Technology PO Box 2320, N-7004 Trondheim, Norway. E-mail: geir.bjorkoy@ntnu.no, phone +47 92243387 or Anders Sundan, Norwegian University of Science and Technology PO Box 2320, N-7004 Trondheim, Norway. E-mail: anders.sundan@ntnu.no, phone +47 72825339.

**Supplemementary Figure legends**.

**Supplementary Figure S1**. **The effect of cysteine and GSH supplement on bortezomib-induced cytotoxicity in myeloma cell lines**. **(A)** INA-6 or ANBL-6 cells were treated with 4 nM bortezomib (Bz) for 24 hours. Where indicated, cells were incubated in the presence of 1 mM cysteine (Cys), Alanine (Ala), GSH or Minimal Essential Medium (MEM, 1X). Experiments were performed at least three times for INA-6 and at least two times for ANBL-6. Cell viability was analyzed using AnnexinV-PI staining as described in the methods-section. Representative results from at least three independent experiments are shown. INA-6 **(B)** and ANBL-6 cells **(C)** were grown for 24 hours in RPMI or diluted 1:1 in HANKS balanced salt solution (denoted HBSS), treated with 4 nM bortezomib, and supplemented with 1 mM cysteine or GSH. Cell viability was analyzed as in (A). Results are mean and SD from at least three independent experiments. Asterisks indicate statistically significant differences (2way ANOVA, Turkey’s multiple comparisons test, p<0,05). **(D)** The indicated cell lines were treated with 4 nM bortezomib and 1 mM cysteine (Cys). Cell viability was analyzed as in (A). Data are average and standard deviations from two independent experiments.

**Supplementary Figure S2. Sulfasalazine potentiated bortezomib-induced cytotoxicity in primary myeloma cells**.

**(A-C)** Isolated CD138^+^ plasma cells from three different myeloma patients were treated for 3 days with bortezomib (Bz) alone or in combination with 0,25 mM sulfasalazine (SASP). Cell death was measured by the ScanR microscope as described in the methods-section. Data are mean and SD for duplicates in one experiment. IC50 (nM) was calculated using non-linear regression. Extra sum-of-squares F-test was used to test for significant shifts in IC50 (p<0,05). Sulfasalazine-treatment induced a significant decrease in IC50 of bortezomib for two of the patients (A, B).

**Supplementary Figures**.

**Supplementary Figure S1**.

**
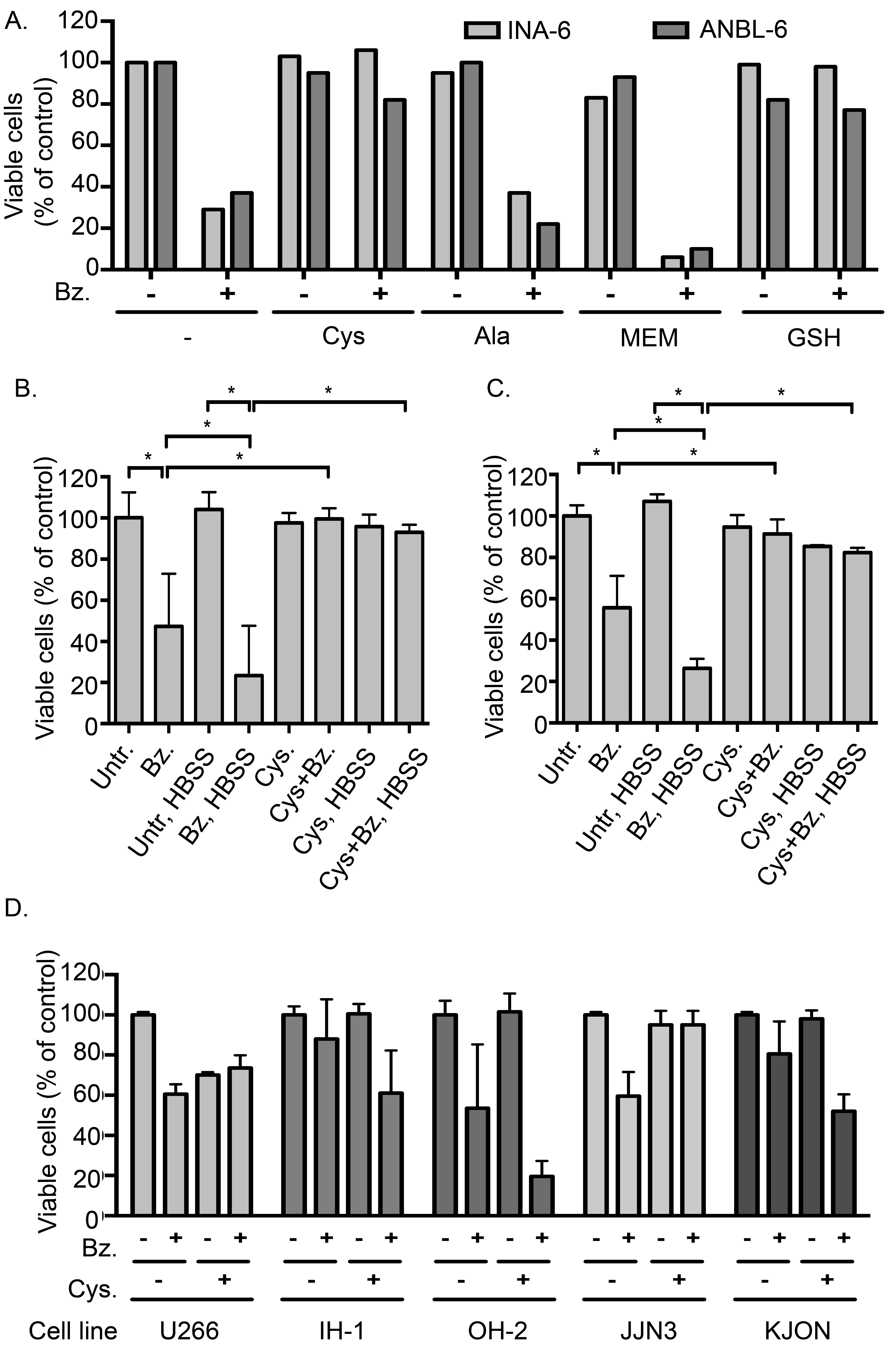
**

**Supplementary Figure S2.**


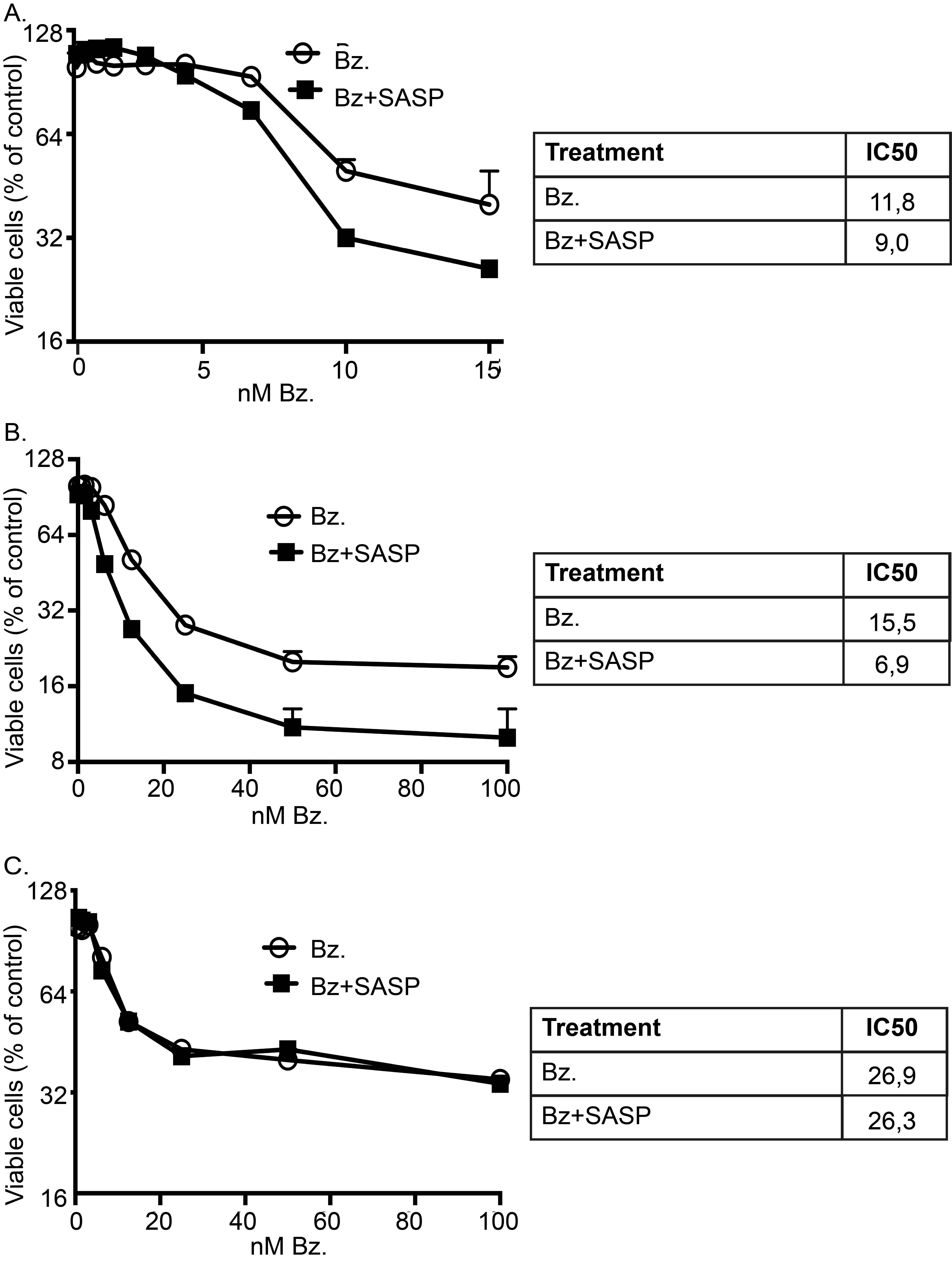


**Supplementary Tables**.

**Supplementary Table S1: Analysis of bortezomib effects using gene expression microarray analysis**. Detailed information of fold change ratios and p-values to the genes showed in the heatmap in figure 2A. List of genes upregulated **(A)** and downregulated **(B)** by bortezomib (Bz) treatment (p<0.05), and significantly reversed when treated with the combination bortezomib+GSH (bortezomib/bortezomib+GSH < 0.05), with cutoff log FC ratio bortezomib vs DMSO >0.5 for the upregulated genes and log FC >0.4 for the downregulated genes. Results are expressed as fold change ratio between the different conditions. B/D: Bortezomib/DMSO. B+GSH/D: Bortezomib+Glutathione/DMSO.

| **A. Up-regulated genes** | | |  |  |
| --- | --- | --- | --- | --- |
|  | **Fold change** | | **p-values** | |
| **Gene** | **B/D** | **B+GSH/D** | **B/D** | **B+GSH/D** |
| HSPA1A | 2,6 | 1,3 | 5,9E-07 | 0,079 |
| DDIT4 | 2,4 | 1,6 | 3,8E-09 | 1,19E-05 |
| DDIT3 | 2,1 | 1,2 | 1,5E-07 | 0,078 |
| CHAC1 | 2,0 | 1,3 | 1,6E-04 | 0,200 |
| HSPA1B | 1,9 | 1,1 | 4,3E-04 | 0,730 |
| BRF2 | 1,9 | 1,4 | 4,2E-07 | 0,001 |
| TSC22D3 | 1,6 | 1,3 | 3,6E-05 | 0,078 |
| ATF3 | 1,6 | 1,4 | 3,6E-05 | 0,005 |
| SEMA3E | 1,5 | 1,0 | 4,2E-02 | 0,992 |
| HAT1 | 1,5 | 1,1 | 7,1E-03 | 0,871 |
| PELO | 1,5 | 1,3 | 1,1E-03 | 0,115 |
| CHRNA5 | 1,5 | 1,1 | 2,1E-02 | 0,858 |
| EIF2AK3 | 1,5 | 1,2 | 2,7E-04 | 0,115 |
| FKBP14 | 1,5 | 1,1 | 1,4E-02 | 0,723 |
| SRXN1 | 1,5 | 1,2 | 1,5E-04 | 0,266 |
| YWHAE | 1,5 | 0,9 | 3,9E-02 | 0,805 |
| QRFPR | 1,5 | 1,0 | 4,3E-02 | 0,938 |
| UBXN4 | 1,5 | 1,0 | 7,8E-03 | 0,974 |
| LOC100129269 | 1,5 | 1,0 | 1,0E-02 | 0,895 |
| ZFAND2A | 1,5 | 1,2 | 2,2E-04 | 0,062 |
| XRCC2 | 1,5 | 1,1 | 2,6E-02 | 0,849 |
| LOC388692 | 1,4 | 1,0 | 3,8E-02 | 0,936 |
| RHBDD3 | 1,4 | 1,0 | 2,4E-02 | 0,966 |
| ORC3 | 1,4 | 1,1 | 3,8E-03 | 0,734 |
| GCLM | 1,4 | 1,0 | 2,0E-03 | 0,990 |
| DUSP19 | 1,4 | 1,1 | 2,4E-02 | 0,800 |
| EXO5 | 1,4 | 1,1 | 3,8E-02 | 0,877 |
|  |  |  |  |  |
|  |  |  |  |  |
| **B. Down-regulated genes.** | | |  |  |
|  | **Fold change** | | **p-values** | |
| **Gene** | **B/D** | **B+GSH/D** | **B/D** | **B+GSH/D** |
| ID2 | 0,7 | 0,9 | 3,6E-05 | 0,204 |
| SLC25A22 | 0,7 | 1,0 | 4,4E-02 | 0,930 |
| SPRY1 | 0,7 | 0,8 | 3,6E-05 | 0,006 |
| NFKBIA | 0,7 | 1,2 | 1,3E-02 | 0,579 |
| INSIG1 | 0,7 | 1,0 | 8,1E-03 | 0,999 |
| PLD6 | 0,7 | 0,9 | 1,4E-04 | 0,201 |
| CDC20 | 0,7 | 0,9 | 4,0E-02 | 0,891 |

**Supplementary Table S2. Functional annotation of genes whose bortezomib-induced up-regulation is attenuated by GSH**. Genes were analyzed by their Biological Process Gene Ontology (GO) annotations using the DAVID Functional Annotation Tool. This resulted in 25 annotation clusters significantly enriched. The 4 most significant are shown in this table.

**(A)** Annotation cluster 1. Enrichment Score: 3.48

| **Term** | **Count** | **%** | **P-value** | **Benjamini** |
| --- | --- | --- | --- | --- |
| **GO:0006986: response to unfolded protein** | 7 | 4.96 | 1.1E-5 | 1.0E-2 |
| **GO:0034976: response to endoplasmic reticulum stress** | 5 | 3.54 | 9.45E-5 | 2.9E-2 |
| **GO:0006984: ER-nuclear signaling pathway** | 5 | 3.55 | 1.1E-4 | 2.4E-2 |
| **GO:0051789: response to protein stimulus** | 7 | 4.96 | 1.1E-4 | 2.0E-2 |
| **GO:0034620: cellular response to unfolded protein** | 4 | 2.84 | 4.2E-4 | 6.3E-2 |
| **GO:0030968: endoplasmic reticulum unfolded protein response** | 4 | 2.84 | 4.2E-4 | 6.3E-2 |
| **GO:0006983: ER overload response** | 3 | 2.13 | 2.2E-3 | 1.8E-1 |
| **GO:0010033: response to organic substance** | 11 | 7.80 | 3.2E-2 | 5.7E-1 |

**(B)** Annotation cluster 2. Enrichment Score: 2.24

| **Term** | **Count** | **%** | **P-value** | **Benjamini** |
| --- | --- | --- | --- | --- |
| **GO:0034976: response to endoplasmic reticulum stress** | 5 | 3.54 | 9.45E-5 | 2.9E-2 |
| **GO:0006984: ER-nuclear signaling pathway** | 5 | 3.55 | 1.1E-4 | 2.4E-2 |
| **GO:0006983: ER overload response** | 3 | 2.13 | 2.2E-3 | 1.8E-1 |
| **GO:0052547: regulation of peptidase activity** | 4 | 2.84 | 2.3E-2 | 4.9E-1 |
| **GO:0051336: regulation of hydrolase activity** | 7 | 4.96 | 3.3E-2 | 5.7E-1 |
| **GO:0043281: regulation of caspase activity** | 3 | 2.13 | 1.1E-1 | 8.0E-1 |
| **GO:0052548: regulation of endopeptidase activity** | 3 | 2.13 | 1.2E-1 | 8.0E-1 |

**(C)** Annotation cluster 3. Enrichment Score: 2.19

| **Term** | **Count** | **%** | **P-value** | **Benjamini** |
| --- | --- | --- | --- | --- |
| **GO:0044092: negative regulation of molecular function** | 10 | 7.09 | 6.0E-4 | 7.7E-2 |
| **GO:0043086: negative regulation of catalytic activity** | 8 | 5.67 | 3.5E-3 | 2.4E-1 |
| **GO:0051346: negative regulation of hydrolase activity** | 4 | 2.84 | 4.8E-3 | 2.6E-1 |
| **GO:0010466: negative regulation of peptidase activity** | 3 | 2.13 | 9.6E-3 | 3.3E-1 |
| **GO:0052547: regulation of peptidase activity** | 4 | 2.84 | 2.3E-2 | 4.9E-1 |
| **GO:0051336: regulation of hydrolase activity** | 7 | 4.96 | 3.3E-2 | 5.7E-1 |

**(D)** Annotation cluster 4. Enrichment Score: 1.57

| **Term** | **Count** | **%** | **P-value** | **Benjamini** |
| --- | --- | --- | --- | --- |
| **GO:0006979: response to oxidative stress** | 7 | 4.96 | 1.1E-3 | 1.1E-1 |
| **GO:0055114: oxidation reduction** | 7 | 4.96 | 3.0E-1 | 9.5E-1 |

**Supplementary Table S3**. **Functional annotation of genes whose bortezomib-induced down-regulation is attenuated by GSH**. Genes were analyzed as in Supplementary Table 1. This resulted in 23 annotation clusters significantly enriched. The 4 most significant are shown in this table.

**(A)** Annotation cluster 1. Enrichment Score: 2.35

| **Term** | **Count** | **%** | **P-value** | **Benjamini** |
| --- | --- | --- | --- | --- |
| **GO:0006350: transcription** | 31 | 22.1 | 5.6E-4 | 9.2E-2 |
| **GO:0006351: transcription, DNA-dependent** | 8 | 5.71 | 8.0E-3 | 3.7E-1 |
| **GO:0032774: RNA biosynthetic process** | 8 | 5.71 | 8.6E-3 | 3.6E-1 |
| **GO:0006366~transcription from RNA polymerase II promoter** | 7 | 5.0 | 1.0E-2 | 3.8E-1 |

**(B)** Annotation cluster 2. Enrichment Score: 2.10

| **Term** | **Count** | **%** | **P-value** | **Benjamini** |
| --- | --- | --- | --- | --- |
| **GO:0033554: cellular response to stress** | 14 | 10.0 | 4.7E-4 | 1.5E-1 |
| **GO:0006974: response to DNA damage stimulus** | 10 | 7.14 | 2.6E-3 | 2.8E-1 |
| **GO:0006259: DNA metabolic process** | 9 | 6.43 | 4.5E-2 | 6.2E-1 |
| **GO:0006281: DNA repair** | 6 | 4.29 | 7.2E-2 | 7.1E-1 |

**(C)** Annotation cluster 3. Enrichment Score: 1.82

| **Term** | **Count** | **%** | **P-value** | **Benjamini** |
| --- | --- | --- | --- | --- |
| **GO:0051301: cell division** | 10 | 7.14 | 5.1E-4 | 1.2E-1 |
| **GO:0001889: liver development** | 3 | 2.14 | 6.5E-2 | 6.9E-1 |
| **GO:0031099: regeneration** | 3 | 2.14 | 1.0E-1 | 7.7E-1 |

**(D)** Annotation cluster 4. Enrichment Score: 1.25

| **Term** | **Count** | **%** | **P-value** | **Benjamini** |
| --- | --- | --- | --- | --- |
| **GO:0009628: response to abiotic stimulus** | 8 | 5.71 | 2.6E-2 | 5.0E-1 |
| **GO:0042127: regulation of cell proliferation** | 12 | 8.57 | 4.4E-2 | 6.2E-1 |
| **GO:0008285: negative regulation of cell proliferation** | 6 | 4.29 | 1.5E-1 | 8.4E-1 |
