# Supplementary figures and images for: Intracellular glutathione determines bortezomib cytotoxicity in multiple myeloma cells
Source: Blood Cancer J. 2016 Jul 15;6(7):e446–. doi: 10.1038/bcj.2016.56 (PMC5141348; doi:10.1038/bcj.2016.56)

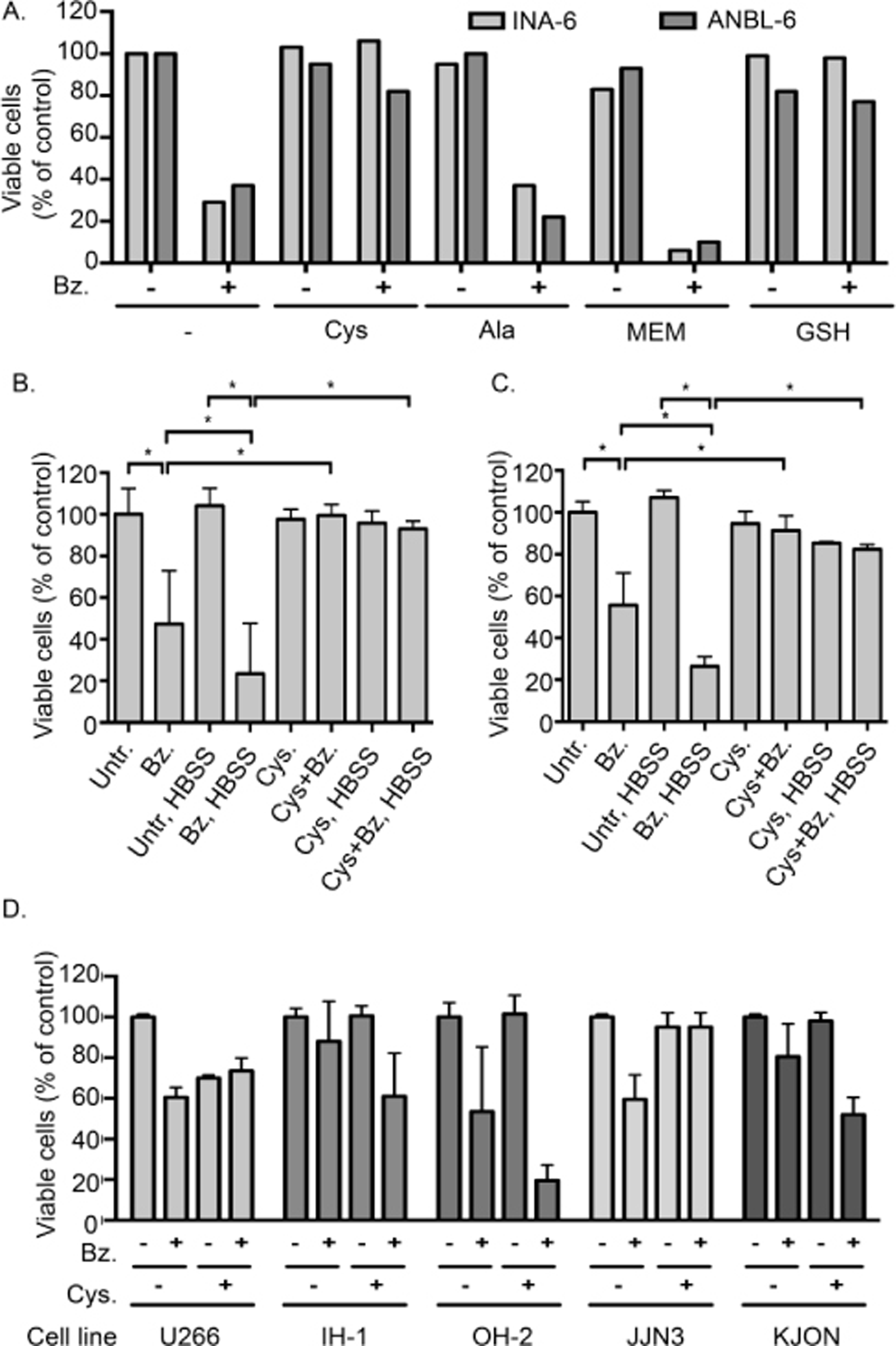

Supplement: Supplementary Figure S1 [file bcj201656x2.tif]

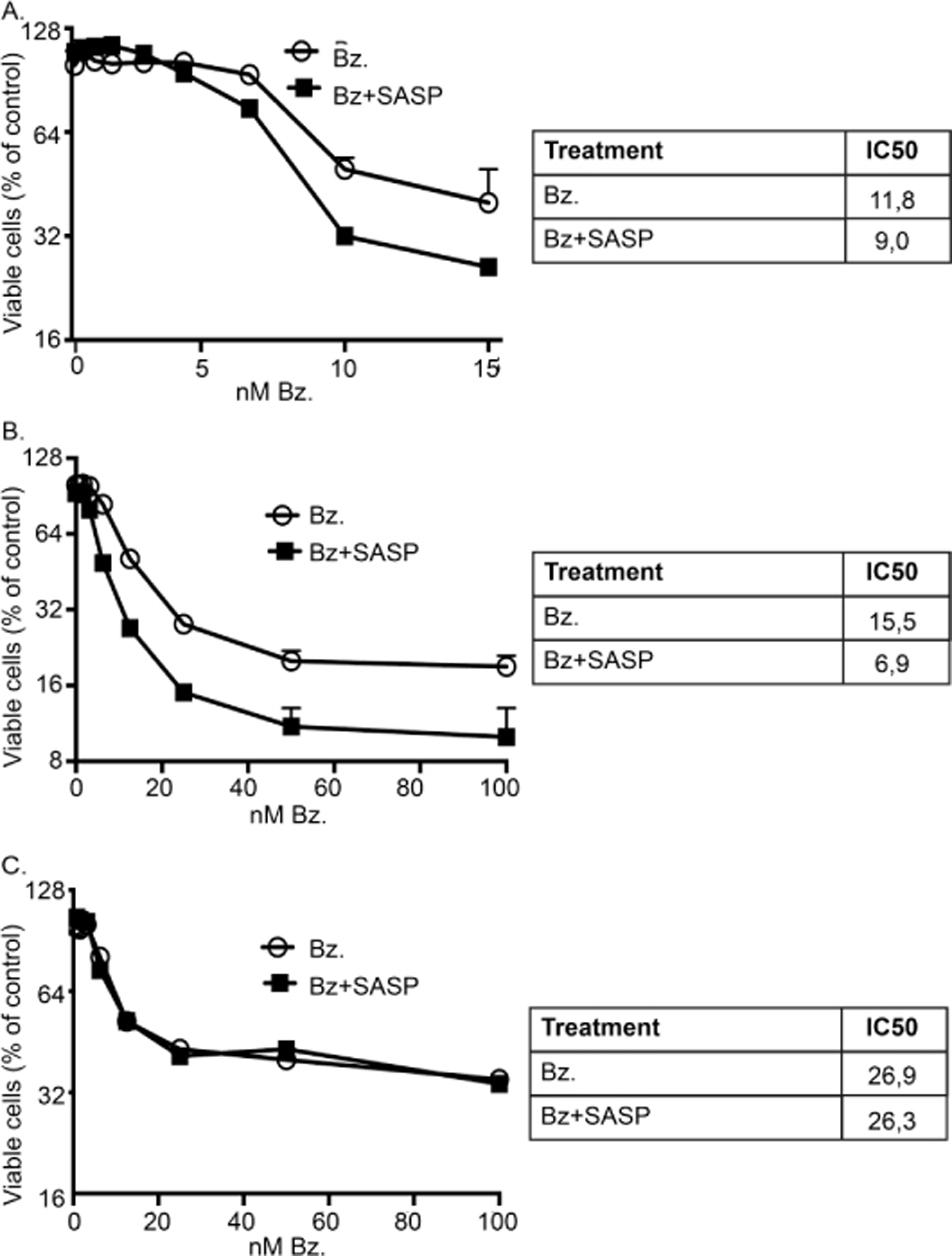

Supplement: Supplementary Figure S2 [file bcj201656x3.tif]
